# Editorial: Age-related changes in auditory perception

**DOI:** 10.3389/fpsyg.2022.986586

**Published:** 2022-08-08

**Authors:** Leah Fostick, Bruce A. Schneider

**Affiliations:** ^1^Department of Communication Disorders, Ariel University, Ariel, Israel; ^2^Department of Psychology, University of Toronto, Toronto, ON, Canada

**Keywords:** auditory perception, aging, bottom-up, top-down, speech perception

Auditory sensory systems have evolved to enable us to detect, locate, identify, and comprehend the various sound sources in our environment, in the form of auditory streams. In turn, the information available in these auditory streams has to be integrated with our world knowledge to determine how we should respond to our immediate environment, and to select or focus our attention on the auditory streams that are important to our short- or long-term survival. In other words, navigating in the real world requires both bottom-up processing of information to arrive at an accurate representation of our environment, and top-down control over the upward flow of information to focus attention on aspects of the environment that are critical to our survival. Hence, age-related changes in sensory and perceptual processes are quite likely to affect cognitive processing (e.g., speech comprehension), not only because such changes may degrade the perceptual representations of stimuli, but also because the degradation of the sensory information is likely to increase the demand on resources that are also required for efficient cognitive processing of the incoming information.

This present Research Topic of papers addresses a number of critical issues with regard to how aging might affect the pattern of interactions between bottom-up and top-down processes that are involved in the extraction of information about the world we live in. As a starting point, it should be noted that there is a huge body of evidence that the bottom-up processing of auditory information deteriorates with age. Pure-tone thresholds, temporal discriminability, the sensitivity of individuals to interaural cues, etc., decline with age. As a result, older individuals necessarily have to rely more than do younger individuals on higher-order, cognitive processes to extract information from the auditory signal when that signal is embedded in soundscapes consisting of a multitude of other auditory sources (for a review, see Schneider et al., 2010). Hence, we would expect that older adults who score high with respect to those cognitive abilities believed to be associated with the processing of speech would have better basic auditory capabilities that those older adults scoring lower with respect to those cognitive abilities. In this Research Topic of papers, Humes et al. found significant correlations between visually-assessed working memory and 7 of 8 tests of Basic Auditory Capabilities (TBAC) in a sample of 115 older adults, indicating that higher-order, age-related changes in cognitive level abilities can affect lower level processing of the acoustic scene. In other words, older adults with good working memories are better at extracting the bottom-up information in the auditory signal that allows them to detect, locate, and process attended sound sources.

The facilitative effect on cognitive-level processes, such as working memory on speech understanding, raises the question as to whether there are any age-related differences in the contribution of the cognitive abilities to speech understanding. Tamati et al. (this Research Topic) looked for age-related differences in the relative contributions of bottom-up and top-down processes in the perception of heard speech distorted by noise vocoding. They presented younger and older listeners with sentences preceded by a visual lexical prime that was ortho-graphically identical to that of the vocoded sentences or with a prime consisting of nonsense words. In addition, the lexical frequency and neighborhood density of the target words in the vocoded sentences were manipulated. Interestingly, although there were significant effects of both bottom-up (noise-vocoding) and top-down (priming, lexical frequency, neighborhood density) in the expected direction, no age-related differences were found, suggesting that the contribution of working memory to speech perception in this situation was equivalent in younger and older listeners.

The complexity of the interaction between top-down and bottom-up processes is illustrated in a third paper in this Research Topic. Weissgerber et al. found that measures on a German language age-standardized test of cognitive ability (Dem Tect) were correlated with speech perception in noise, a correlation that disappeared when corrected for high-frequency hearing loss. They note that a high-frequency hearing loss could have produced lower scores on the Dem Tect test which included acoustically presented test items that could have been misheard due to a high-frequency hearing loss. This result, along with a study by Fullgrabe (2020) that found that simulated hearing loss reduced scores on cognitive tests involving acoustically-presented material in young listeners, indicate that assessment of the higher-order cognitive processes thought to be important in speech perception tasks, can be affected by age-related changes in basic auditory processes. In addition, Zaltz and Kishon-Rabin (this Research Topic) found that even when a cognitive test does not involve aurally presented material, there are complex interactions between tests of basic auditory abilities and cognitive capacities. Specifically, these investigators found that the ability of older adults to take advantage of differences in fundamental frequency and formant structure to discriminate among different voices was related to both hearing sensitivity and a measure of cognitive ability based on a visual test (Trail Making Test).

Shvartzman et al. (this Research Topic), in addition to confirming the importance of the interaction of bottom-up and top-down processes in speech perception, also suggest that individual differences in the ability to rapidly reorganize perceptual processes to respond appropriately to a consistent set of auditory features of a sound source (perceptual learning), are correlated with performance in some types of difficult listening situations such as the processing of rapid speech. This suggests that individuals who are capable of rapid perceptual learning, are better able to adjust to the idiosyncrasies of a person's speech, thereby permitting them to adjust their speech-processing mechanisms to be better able to function in a complex auditory scene, especially one where they are exposed for the first time to a new speaker.

The preceding studies demonstrate that there are complex interactions between basic sensory processes and a number of cognitive processes involved in processing speech. This raises the question of how we might go about dissecting the nature of these processes. One way is to focus on the ability of an individual to make use of the information provided by a basic auditory process in performing a higher-order task where one might expect the performance of the higher-order task to be critically dependent on the information provided by a specific lower-order auditory ability. The study by Szelag et al. is a nice example of how limitations on a lower-level, bottom-up process, can affect the ways in which a higher-order, cognitive task is conducted. The lower-level task in this study was a temporal order judgment. After categorizing individuals into either low or high performers on this temporal order task, they then assessed how they performed a higher-order, but still temporally-based task, that might be expected to be affected by the individual's degree of lower-order temporal discriminability. In the higher-order task, the listener was presented with a series of clicks where the inter-click interval was fixed. The listener was instructed to mentally create a beat structure for this sequence by mentally accentuation some of the beats. These investigators found that the strategies used by participants to create a beat structure depended on their lower-order temporal order judgments, indicating that the strategies that listeners used in performing higher-order auditory tasks are conditional upon the lower-order processing capacities that might be useful in performing the task.

Of course, any task that places demands on a cognitive ability, such as working memory, has implications for situations where a person, in addition to attempting to understand speech in a noisy situation, has to simultaneously perform a different task that also draws on this cognitive level ability. The Nitsan et al. eye-tracking study (this Research Topic) found that the way in which older adults processed words presented in noise while performing a secondary task (digit recall) depended on their working memory capacity. The working memory load on the secondary task could be either low (1 digit presented) or high (4 digits presented). On trials in which the target word was correctly identified (by means of eye-tracking), the working-memory load on the secondary task affected the proportion of times the low-working-memory capacity individuals responded correctly. Their performance in the secondary digit recall task dropped significantly from when the working-memory load on the secondary task was low (a single digit) to when it was high (4 digits). No such decline was observed in the high-working-memory individuals. The pattern of eye movements during this task indicated that there were differences between high- and low-working-memory individuals in the ways in which top-down resources were allocated to this task, and that individuals with low working-memory capacity, when faced with a high-working-memory demand on the primary task might be unable to muster sufficient resources to perform well on the secondary task.

Clearly, demonstrating that there is a complex pattern of interaction between top-down and bottom-up processes involved in using auditory information to help us understand and navigate in the real world, doesn't specify precisely how, when, and where in sensory and perceptual processing such interactions take place. What is needed is the ability to examine this process through a moving temporal window to help us understand when and where such interactions occur. A number of studies have begun to use eye-tracking techniques to provide us with a temporal breakdown of this process. For example, Failes and Sommers (this Research Topic) used eye-tracking to identify age-related differences in the degree to which younger and older adults differed with respect to how preceding sentential context affected the correct identification of the final word in a spoken sentence. In some sentences, the preceding sentential context supported the sentence final word, in other sentences the preceding sentential context suggested a phonological competitor, while in a third type of sentence, the context prior to the sentence final word could not be readily used to predict the sentence-final word. Four images of objects were shown on a screen prior to the presentation of the test sentence with one of the objects corresponding to the sentence final word, another to a phonological competitor, along with two objects used as foils. By comparing the time-course of eye movements among these objects, they were able to identify intriguing differences between younger and older subjects with respect to how and when context influenced an individual's pattern of fixations during the presentation of sentences, supporting the notion that the manner in which top-down knowledge affects speech perception, can differ with age.

We also need to consider the contribution of non-auditory cues that contribute to speech understanding in everyday environments: namely the importance of visual cues to speech in difficult listening situations. Gordon-Salant et al. (this Research Topic) presented younger and older listeners (with and without hearing losses) with a visual image of the speaker they were attending to and varied the asynchrony of the visual and auditory components of speech. These investigators found that older adults (both with and without hearing loss) had higher thresholds for detecting an asynchrony between visible and audible speech. However, in all three groups, speech perception scores were equally affected by the degree of asynchrony, indicating that the contribution of visible speech to speech perception was the same in younger and older adults (once the signal-to-noise ratio was adjusted to produce equivalent speech recognition scores in all three groups) but that older adults had a higher threshold for detecting an asynchrony between the two. Here, as well as in other studies in this Research Topic, the effectiveness of top-down processes does not appear to be significantly affected by age when speech understanding is the primary task, suggesting that higher-order mechanisms remain effective in aging listeners. However, this does not necessarily mean that the manner in which this knowledge is used in aid of speech perception is the same in younger and older listeners (see Failes and Sommers above).

Spoken language, in addition to conveying semantic information from the talker to the listener also contains emotional information as well. There are two sources of emotional information in speech: the semantic content of the speech that conveys information about an emotional state (e.g., I am really sad about this vs. I am really mad about this); and/or its prosody (the emotional tone of the speech conveyed by suprasegmental information derived from the tone of the speech such as the stress pattern, rhythm, and pitch). In this Research Topic of papers, Dor et al. looked for age-related changes in the ability of listeners to identify the emotional content of the speech when it was being masked by speech-spectrum noise. These investigators found, that although older adults needed a higher signal-to-noise ratio than younger adults, for both age groups, the emotion conveyed by prosody required a smaller signal-to-noise ratio for detection than emotion conveyed by semantic content, and that the percentage of correct detection of emotion increases in the same way as a function of the signal-to-noise ratio for both age groups. This suggests that the cognitive mechanisms responsible for the detection of emotion do not change with age. However, when the semantic emotional content differs from the prosodic emotional content, and the listener was asked to base their judgment on only one of the two conveyers of emotion, there was some evidence that older adults' judgements were more affected by the not-to-be-attended channel than younger adults, indicating potential age-related capacity limitations on top-down resources.

In considering how age affects auditory processing, it is also reasonable to investigate how technological changes have affected our soundscapes (e.g., the increasing importance of broadcast media, the use of sound amplification, surround sound, and immersive environments), and whether older adults are as well-equipped as younger adults to navigate these environments. Russell, in this Research Topic, discusses our limited understanding of how chronological age affects the perception of space, when that perception is based on acoustic cues. This limitation extends to how spatial perception is altered by technological changes in our everyday soundscapes. One of these changes involves the broadcast of the audio and visual components of a scene to remote receivers with the result that delays are occasionally introduced between the audio and visual portions of the broadcasts (for the effects of such delays, see Gordon-Salant et al., this Research Topic). Another instance involves the often-ubiquitous use of surround sound, which removes some of the acoustic cues to the spatial location of a sound source and changes its timbre due to comb filtering. Hence, modern soundscapes can consist of a mixture of well-localized auditorily compact sources, as well as those that have a much more diffuse timbre and are less precisely localized.  Avivi-Reich et al. (this Research Topic) studied the ability of young adults (both native and non-native speakers of English) and older native speakers of English to identify auditory targets in a background of competing sound sources. Both targets and competing masking sources could be either compact or diffuse. They found that aside from the usual signal-to-noise differences between younger and older native listeners, the effects of a difference in timbre between masker and target were the same for these two groups. However, the younger non-native listeners differed from the former two groups in that the young non-native group tended to perceive all four combinations of target and masker timbre equivalently. This suggests that the listener's knowledge of the language affected how well they could make use of timbre differences due to the use of surround sound, whereas their age did not affect their ability to respond to timbre differences.

A common theme that appears to be emerging from the studies in this Research Topic is that, provided that the draw on top-resources is not too extensive (as could happen in dual-task situations), older adults without cognitive impairment are as capable as younger adults in using top-down knowledge and top-down processing abilities to parse the auditory scene, and to extract targeted information from that scene. However, in everyday situations, older adults most likely have to draw more on top-down resources in order to maintain an acceptable level of speech understanding than do younger adults, even when the signal-to-noise ratios are adjusted to equate performance between these two groups. This suggests that listening in everyday situations is more effortful for older than for younger adults, which could result in greater fatigue and a withdrawal from social interactions. Therefore, there exists a need in the clinic to assess the degree of effort involved in listening in noisy settings. In laboratory settings, dual-task paradigms are typically used to assess listening effort (see the Nitzen et al. study, this Research Topic, for an example of the use of a dual-task situation). Neeman et al. (this Research Topic) have developed and tested a relatively simple test of listening effort using equipment that would be found in audiological settings. They gave this test to a sample of both young and middle-aged adults. In this task, they adjusted the signal-to-noise ratio to obtain individual speech reception thresholds of 80% correct while measuring the cost of the dual-task on the secondary task. They found listening-effort effects in both age groups. However, the cost of the secondary task was greater in the middle-aged listeners than in the younger listeners, indicating that listening effort increases with age, and that this increased effort can be assessed in audiological settings, and allow the audiologist to address the patients' concerns by discussing with them the cost of listening in noisy environments and ways in which such costs can be reduced (e.g., use of assistive devices such as directional microphones, etc.).

Finally, there is the question of how effective certain interventions are in improving the quality of the lives of older persons with hearing impairment, such as cochlear implant users. In this issue, Brumer et al. evaluated the health-related quality of life of individuals with cochlear implants. These investigators found that bimodal and bilateral cochlear implant users who were better able to function in noisy environments experienced a higher degree of life-satisfaction, as measured by the Glasgow Benefit Inventory. More studies are needed on how these and other types of interventions can improve the quality of life of individuals experiencing communication difficulties.

This Research Topic of papers clearly indicates the complexities involved in utilizing acoustic cues to extract information that is not only important to our degree of life-satisfaction but even to our survival. Understanding speech, for example, requires the integration of information registered on the cochlear with information coming in from other senses (primarily vision) and from our stored world knowledge. The studies in this Research Topic contribute to our understanding of this extremely complex process. They also illustrate that there is: (1) much more to learn with respect to how speech understanding is accomplished in the noisy environments typical of everyday life, and (2) how we can utilize the information coming from such studies to improve auditory environments, and to help those with diminished auditory and/or cognitive abilities to function in difficult listening situations.

## Author contributions

LF and BS contributed to the conception of the Research Topic. Both authors contributed to the manuscript and approved the submitted version.

## Conflict of interest

The authors declare that the research was conducted in the absence of any commercial or financial relationships that could be construed as a potential conflict of interest.

## Publisher's note

All claims expressed in this article are solely those of the authors and do not necessarily represent those of their affiliated organizations, or those of the publisher, the editors and the reviewers. Any product that may be evaluated in this article, or claim that may be made by its manufacturer, is not guaranteed or endorsed by the publisher.
